# Modeling Seasonal Influenza Transmission and Its Association with Climate Factors in Thailand Using Time-Series and ARIMAX Analyses

**DOI:** 10.1155/2015/436495

**Published:** 2015-11-18

**Authors:** Sudarat Chadsuthi, Sopon Iamsirithaworn, Wannapong Triampo, Charin Modchang

**Affiliations:** ^1^Department of Physics, Faculty of Science, Naresuan University, Phitsanulok 65000, Thailand; ^2^Department of Disease Control, Ministry of Public Health, Tivanond Road, Nonthaburi 11000, Thailand; ^3^Biophysics Group, Department of Physics, Faculty of Science, Mahidol University, Bangkok 10400, Thailand; ^4^Institute for Innovative Learning, Mahidol University, Nakhon Pathom 73170, Thailand; ^5^Centre of Excellence in Mathematics (CHE), 328 Si Ayutthaya Road, Bangkok 10400, Thailand; ^6^ThEP Center, CHE, 328 Si Ayutthaya Road, Bangkok 10400, Thailand

## Abstract

Influenza is a worldwide respiratory infectious disease that easily spreads from one person to another. Previous research has found that the influenza transmission process is often associated with climate variables. In this study, we used autocorrelation and partial autocorrelation plots to determine the appropriate autoregressive integrated moving average (ARIMA) model for influenza transmission in the central and southern regions of Thailand. The relationships between reported influenza cases and the climate data, such as the amount of rainfall, average temperature, average maximum relative humidity, average minimum relative humidity, and average relative humidity, were evaluated using cross-correlation function. Based on the available data of suspected influenza cases and climate variables, the most appropriate ARIMA(X) model for each region was obtained. We found that the average temperature correlated with influenza cases in both central and southern regions, but average minimum relative humidity played an important role only in the southern region. The ARIMAX model that includes the average temperature with a 4-month lag and the minimum relative humidity with a 2-month lag is the appropriate model for the central region, whereas including the minimum relative humidity with a 4-month lag results in the best model for the southern region.

## 1. Introduction

Influenza, commonly referred to as the flu, is a worldwide respiratory infectious disease that easily spreads from one person to another. Influenza is the cause of approximately 3–5 million cases of severe illness and 250,000–500,000 deaths annually worldwide [1]. The disease is transmitted through the air by coughs or sneezes, creating aerosols containing the virus from infectious individuals. Individuals, who come into contact with or breathe in these aerosols, will likely become infected by the virus [1]. The first reported major global pandemic, known as the “Spanish” influenza, occurred in 1918; it was caused by a novel H1N1 virus subtype [2]. This pandemic was estimated to have cost the lives of 20–40 million people from 1918 to 1919 [3].

In temperate regions, influenza has a seasonal pattern peaking during winter seasons [4], whereas, in tropical regions, the incidence is less likely to be seasonal rather than randomly patterned [5]. The important role of climate in influenza is less understood for tropical regions. However, a marked increase of influenza cases was reported during the rainy seasons, in countries such as Singapore, northeastern Brazil, and French Guiana [5–7]. In Dakar, Senegal, the incidence of influenza peaked during 1996–1998. This peak was attributed to high precipitation, humidity, and temperature [8]. From the laboratory surveillance data in Brazil, Alonso et al. found that temperature and humidity played an important role in driving the influenza epidemic [9]. Influenza incidences were also associated with temperature in tropical countries [10]. Moreover, in warm climates, the recording of environmental variables was shown to increase the ability to predict influenza cases [11]. An experimental study showed that the spread of the influenza virus depends upon both temperature and relative humidity [12, 13]. It was found that aerosolized influenza virus is stable maximally at low relative humidity conditions and moderately stable at high relative humidity [14]. From simulated coughs, influenza viruses maintain infectivity at low relative humidity and are increasingly inactivated at high relative humidity [15].

In Thailand, few reports of the seasonality of influenza outbreaks exist. Influenza cases peaked twice per year in the 11 provinces of Thailand during 2004–2010, as monitored by passive surveillance. A major peak occurred during the rainy season (June–August), and a minor peak occurred during the winter season (October–February) [16]. Climate factors may play an important role in predicting the number of influenza cases. The links between climatic variables and influenza cases in Thailand are also less understood. Using regression analysis, Chumkiew et al. found that the temperature difference and percent of rainfall were associated with the influenza incidences in Nakhon Si Thammarat [17]. However, this research covered only one province in Thailand. Most of the studies in Thailand focused on the relation of the seasonal patterns of influenza outbreaks [18, 19]. Knowledge of the effects of climatic variables on the influenza seasonality in Thailand may be important for developing efficient intervention measures that may help mitigate and/or contain the disease.

Here, we retrospectively analyze the time-series pattern of reported suspected influenza cases in 2 regions of Thailand during the years 2009 to 2014. We used autocorrelation and partial autocorrelation plots to determine the ARIMA model that predicts the future influenza cases with a linear function of time lag values (autoregressive part) plus uncorrelated random variables (moving average part). The relationships between the climate data and reported influenza cases were evaluated using cross-correlation function. We followed previous suggestions [5–11] and investigated each climatic variable (the amount of rainfall, average temperature, average maximum relative humidity, average minimum relative humidity, and average relative humidity) that may influence the influenza cases as input time-series in the ARIMAX model. The most appropriate ARIMAX model for each region is presented, based on the previous data and climate variables.

## 2. Materials and Methods

### 2.1. Data Sources

Data of monthly influenza cases from 2009 to 2014 were extracted from the National Notifiable Disease Surveillance Report of the Bureau of Epidemiology at the Ministry of Public Health [20]. Most positive cases were suspected influenza cases, based on clinical diagnosis made by attending physicians. The clinical criteria for influenza were fever, cough, sore throat, headache, and myalgia. Samples from some suspected influenza cases were then tested using RT-PCR for laboratory confirmation. The suspected influenza cases are reported only from the public hospitals and some private hospitals. In this work, we analyzed only the influenza cases in 2 regions, the central and southern regions, which exhibit more flu cases than other regions in Thailand.

There are three seasons in the central region: the rainy season (mid-May to mid-October), the winter season (mid-October to mid-February), and the summer season (mid-February to mid-May). However, in the southern region, the year is divided into only two seasons: the rainy season (June to February) and the summer season (March to May). Geographically, the southern region is a peninsula located between the Andaman Sea on the west and the South China Sea on the east, whereas the central region has higher latitude.

**Figure 1:**
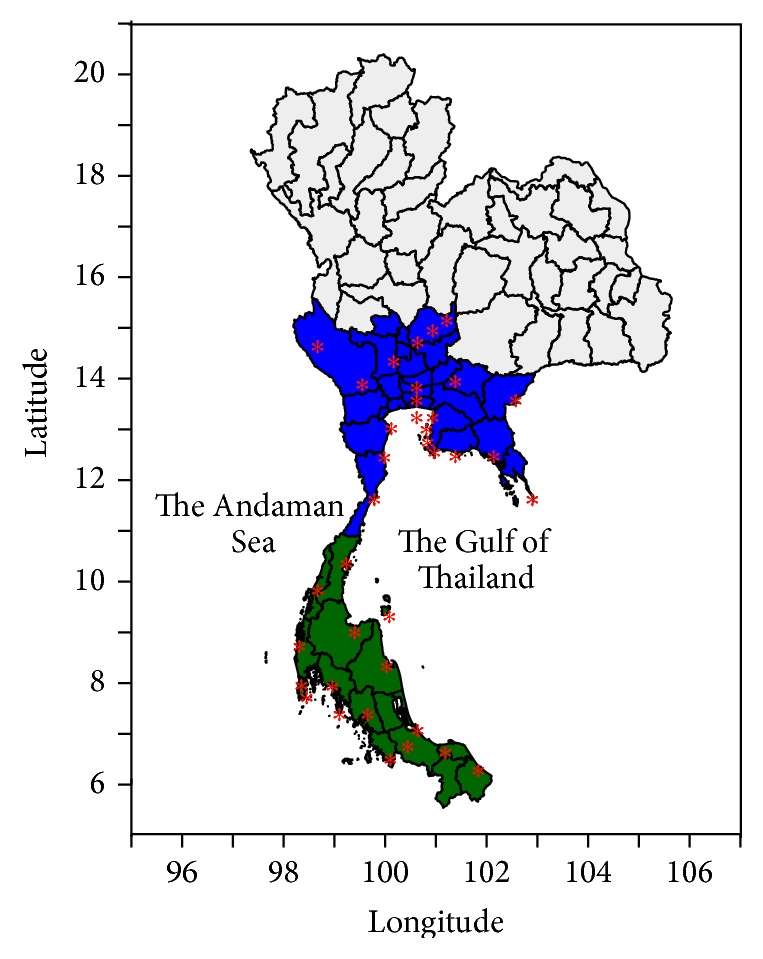
The spatial distribution of weather stations (red stars) in the central (blue area) and southern (green area) regions.

The monthly rainfall, monthly average temperature (*T*_mean_), average maximum relative humidity (RH_max⁡_), and average minimum relative humidity (RH_min⁡_) were obtained from the Research Data Archive (RDA), which is maintained by the Computational and Information Systems Laboratory (CISL) at the National Center for Atmospheric Research (NCAR). The monthly original data can be obtained from the RDA (http://rda.ucar.edu/) in the dataset number ds512.0. We extracted the climate data from 39 weather stations, with 23 stations located in the central region and 16 stations located in the southern region (Figure 1). The climate data extracted from each station were used to determine the average climate value that represented the regional climate data. We also obtained data on rainfall variations in Thailand from the US National Climate Data Center (NCDC). Data is available from 171 stations in Thailand, including 35 stations for the central region and 15 stations for the southern region.

### 2.2. Time-Series Analysis

The time-series data from 2 regions were divided into 2 parts; the first 60 months of data (from 2009 to 2013) was used to calibrate the time-series model, and the last 12 months of data (in 2014) was used to test the model prediction. In this study, we used the autoregressive integrated moving average, ARIMA(*p*, *d*, *q*)(*P*, *D*, *Q*)_*s*_ model. For a complete description of ARIMA analysis, we refer readers to [21]. Briefly, the ARIMA univariate analysis models consisted of 3 subprocesses: (a) autoregression (AR), (b) moving average (MA), and (c) differencing forming a stationary time-series [21–24]. *p*, *d*, and *q* are the orders of the AR, the differencing, and the MA process, respectively, whereas *P*, *D*, and *Q* are the seasonal orders of AR, differencing, and MA process, respectively; *s* is the seasonal period. In the ARIMA model, the predicted influenza cases at time *t*, *Y*_*t*_, have been obtained by applying the weight (*θ*) to the uncorrelated random variables (*e*_*t*_) in the *q*th order. The time-series of *Y*_*t*_ may be written as a linear function of the lag value at *p*th order, as follows:

(1) Yt=ϕ1Yt−1+ϕ2Yt−2+⋯+ϕpYt−p+et−θ1et−1−⋯−θqet−q.

If the time-series were nonstationary, we used log transformation and/or differencing (*W*_*t*_ = *Y*_*t*_ − *Y*_*t*−1_) to remove the nonstationary terms. The *p* and *q* orders can be obtained using the cutoff time lag of autocorrelation function (ACF) and partial autocorrelation function (PACF). If the time-series showed a seasonal pattern with an ACF peak at *s* time lags, then the same procedure was applied to the seasonal ARIMA model. The ARIMA model was examined using the goodness of fit process, in which the residual is likely to be white noise. The coefficients of the model were estimated using the mean square method. Akaike's Information Criterion (AIC) was used to determine the optimal model, which can avoid overparameterization. However, the root mean square error (RMSE) was also used to determine the best fit [11].

The climate variables were used as input time-series in the ARIMAX model. In the case of strongly autocorrelated data, it is difficult to determine the correlation between the time-series of climate and suspected cases. Prewhitening is a useful method to disentangle this strong linear correlation. In this work, the prewhitening process was applied to the climate time-series to avoid autocorrelation with suspected cases time-series. Then, the cross-correlation function (CCF) between the prewhitened climate and suspected influenza case time-series was calculated to identify the significant time lag. Climate time-series that do not show a significant time lag were excluded from the ARIMAX model. The predicted influenza cases at time *t*, *Y*_*t*_, from ARIMAX model were determined by *Y*_*t*_ = *β*_0_ + *βX*_*t*−*d*_ + *Z*_*t*_, where *X*_*t*−*d*_ is the climate series at *d* lags and *Z*_*t*_ is the predicted influenza cases at time *t* and corresponds to the optimal ARIMA(*p*, *d*, *q*)(*P*, *D*, *Q*)_*s*_ model from the previous step. The coefficients of the ARIMAX model were estimated as described above. The multivariate model is used to fit series data and to predict future cases. The associated RMSE was calculated to determine the predicted model. R software, version 3.1.1 (the R foundation for Statistical Computing, https://www.R-project.org/) was used for the time-series analysis and graphic display, and *P* < 0.05 was considered to be statistically significant.

## 3. Results

### 3.1. Climate Patterns

**Figure 2:**
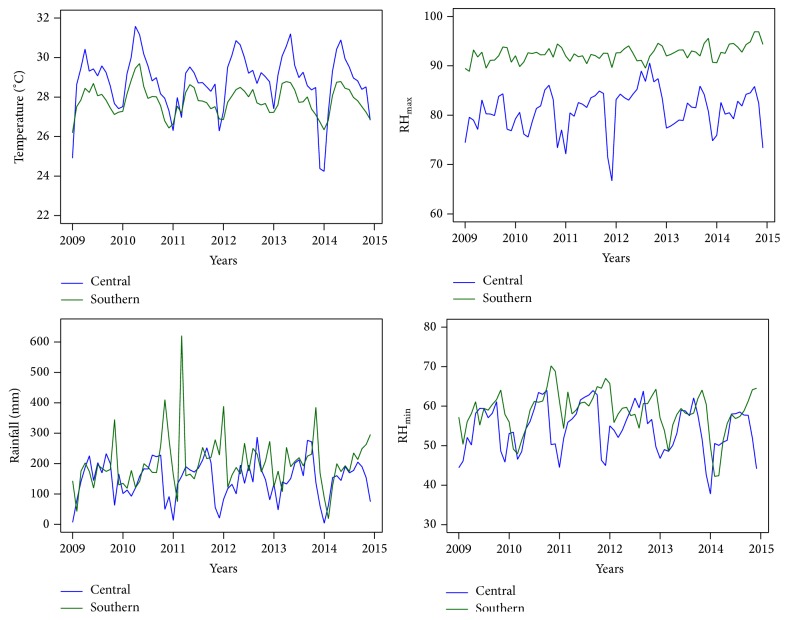
The recorded climate time-series, average temperature (°C), amount of rainfall (mm), average maximum relative humidity (percent), and average minimum relative humidity (percent).

We found that the southern region experienced small temperature variations and that its lowest temperature value is higher than in the central region. The regions also have different climatic variables (Figure 2). The peak temperature in both regions occurred in the summer season. The mean climatic parameters are shown in Table 1. Monthly rainfall in the central and southern regions showed slightly different patterns, with the lowest rainfall level occurring at the end of the year in the central region. We found greater variations in the southern region, with peaks occurring from April to November each year. RH_max⁡_ in the central region is low, whereas RH_max⁡_ in the southern region is higher year-round. This pattern is similar to RH_min⁡_, which shows high value year-round, with the lowest variation in the southern region. RH_min⁡_ and RH_max⁡_ peaks occurred midyear.

### 3.2. ARIMA Model

**Figure 3:**
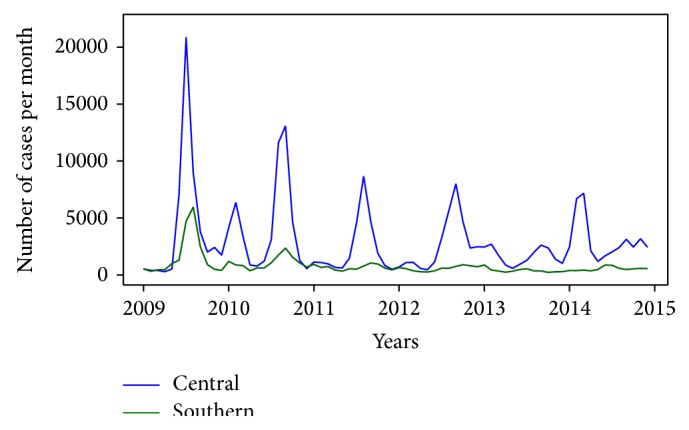
Time-series for influenza cases in the central and southern regions.

The monthly number of suspected influenza cases in the central and southern regions during the period of 2009–2014 is shown in Figure 3. We found that the incidences in the central region were higher than in the southern region and that the case time-series in both regions tentatively decrease over time. We also found that the cases time-series peak once each year.

We hypothesized that climate factors are correlated with suspected influenza time-series. We identified the cross-correlations of climate factors with a time lag of max 4 months [25, 26] for both regions, as shown in Tables 2 and 3. We found that the case time-series in the central region was significantly correlated with rainfall variables at lag of 0–4 months; average temperature at lags 3 and 4; maximum relative humidity at lags 0 and 1; minimum relative humidity at lags 0–2; and average relative humidity at lags 0–2. However, in the southern region, we found significant correlations only with average temperature at lags 3 and 4 and maximum relative humidity at lag 1. We also fitted the suspected influenza data with several univariate ARIMA(*p*, *d*, *q*)(*P*, *D*, *Q*)_*s*_ models using different orders. The best models for each region are shown in Tables 4 and 5 as model 1. We then tested several seasonal ARIMAX models with one or more climate factors at significant lags to find the most appropriate models.

**Figure 4:**
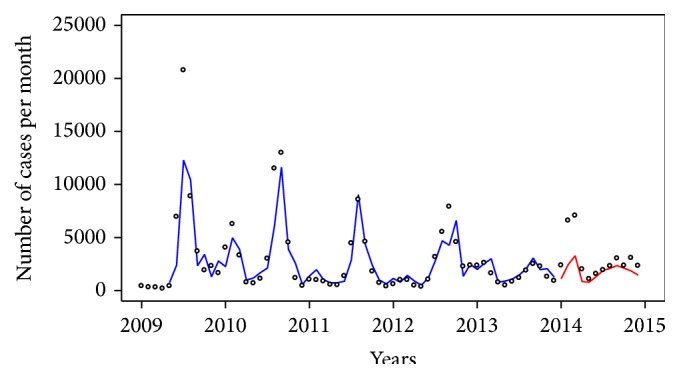
Fitted (blue line) and predicted (red line) values from model 17 (ARIMA(1, 0, 2)(1, 0, 0)_12_ with average temperature (lag 4) and minimum relative humidity (lag 2)) compared with influenza cases (dot) in the central region.

In the central region, we found several significant multivariate models, as shown in Table 4. We first tested the ARIMAX model for one climate variable as input series. The results show that model 10 with minimum relative humidity at lag 1 had the smallest AIC and smallest fitted RMSE. However, model 10 also had the highest predicted RMSE. Model 11 with minimum relative humidity at lag 2 had the smallest predicted RMSE. We further screened the ARIMAX models including two or three climate factors for coefficients presenting highest significance. For three climate factors, no model was found to improve the AIC, fitted or predicted RMSE (data not shown). When including two climatic factors, we found that model 16 with the average temperature at lag 4 and minimum relative humidity at lag 1 has the smallest AIC but also shows the highest predicted RMSE. The difference in AIC for models 16 and 17 is about 3%. However, model 17 showed smallest fitted and predicted RMSE and a higher *P* value coefficient for *T*_mean_ and RH_max⁡_. Therefore, model 17 (ARIMAX(1, 0, 2)(1, 0, 0)_12_ with *T*_mean_ at lag 4 and RH_min⁡_ at lag 1) is in our view the most appropriate for use.  Figure 4 shows the fit and prediction results and concludes that this model can roughly demonstrate the tendency for future cases.

**Figure 5:**
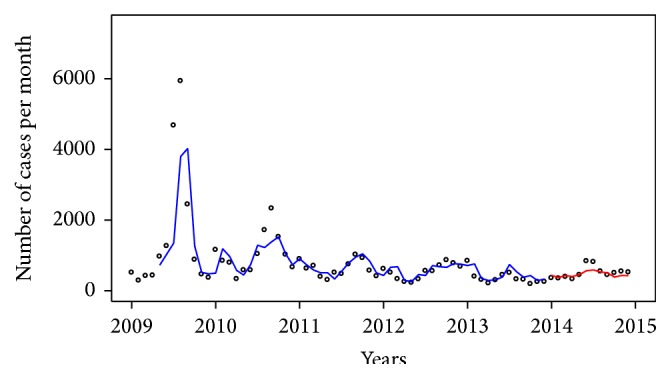
Fitted (blue line) and predicted (red line) values from model 3 (ARIMA(1, 0, 2)(0, 0, 1)_12_ with average temperature (lag 4)) compared with influenza cases (dot) in southern region.

For the southern region, model 3 with an average temperature at lag 4 had the smallest AIC. This model also had the smallest predicted RMSE. However, this model demonstrated a high fitted RMSE, whereas model 2 had the smallest RMSE for prediction (Table 5). For two climate factors, model 6 incorporates *T*_mean_ at lag 4 and RH_min⁡_ at lag 1 and showed values of AIC and RMSE higher than that of model 3, though it had a lower *P* value. The difference of fitted RMSE for models 3 and 5 was less than 1%, but the difference of predicted RMSE was about 15%. Hence, model 3 (ARIMAX(1, 0, 2)(0, 0, 1)_12_ with *T*_mean_ at lag 4) which had the smallest RMSE and smallest AIC was selected for monitoring and prediction. The fitting result is shown in Figure 5.

## 4. Discussion

Suspected influenza cases (exceeding approximately ten thousand cases every year) have been reported by the Bureau of Epidemiology, Thai Ministry of Public Health, on a monthly basis. This study was conducted to provide information about the seasonality of influenza and the impact of climatic variables in 2 geographical regions in Thailand. In the final multivariate model, we found that the best model for fitted and predicted influenza cases for the central region includes average temperature and minimum relative humidity; in the southern region, the best model includes average temperature only.

In this study, we explained the different correlations between the central and southern regions by the differences in climatic conditions of these two regions. In the southern region, the year is divided into two seasons: the rainy season, which lasts for 9 months, and the summer season lasting for 3 months. In the central region, seasons comprise the rainy, winter, and summer seasons, each lasting for 4 months. People in the southern region are exposed to high and relatively constant humidity all year round. Therefore, one could expect people in the southern region to be less prone to contract the influenza virus due to variations of relative humidity than people in the central region. From cross-correlation analysis, we found that only maximum relative humidity correlated with suspected influenza cases in the southern region, whereas all of the maximum, minimum, and average relative humidity correlated with suspected influenza cases in the central region.

In general, an association between influenza cases and local environmental factors, such as humidity and temperature, were found in temperate zones. Low relative humidity increases the rate of infection in guinea pig models, whereas high relative humidity blocks transmission [12]. Low influenza virus survival occurred at high temperatures, and no transmission was detected at temperatures higher than 30°C [12, 13]. Based on simulated coughs, the results also showed inactivation of the virus at higher relative humidity after coughing [15]. However, the findings of this study contradict those of previous research. In the central and southern regions, the influenza peaks occurred during the rainy season which is characterized also by high average temperatures. We can assume that the climate factors may have less influence on airborne transmission in tropical regions [27]. The time-series for the influenza cases may be associated with a minimum relative humidity of 38–64% and average temperatures of 24–32°C for the central and with average temperatures of 26–30°C for the southern regions, respectively. Our models suggest that relative humidity is associated with influenza transmission in the central region. This finding is consistent with previous studies performed in other tropical countries. For example, a negative correlation between the relative humidity and the influenza incidence rate as obtained by the ARIMA model was found in Brisbane and Singapore during 2000–2007 [27]. Furthermore, the logistic regression model for the influenza transmission in subtropical Guatemala with a relative humidity similar to the central region in Thailand also presented a negative correlation with humidity [28].

A positive correlation with temperature was found in this study. This finding is consistent with the study made in west-central El Salvador [28]. The average temperatures in both regions of Thailand are also corresponding to those in west-central El Salvador, which has average temperatures of 25–29°C. In the study [28], it was suggested that temperature may be a proxy for other factors. In a contact transmission experiment, Lowen et al. found that titers from exposed guinea pigs increase at similar patterns at 30°C and 20°C and at both 20% and 80% relative humidity [13]. The amount of virus shed at 30°C and 20°C was not markedly different, which indicates that the rate of transmission did not decrease with temperature [13]. These results correspond to the humid-rainy conditions in tropical climates [26].

In this work, we found that the amount of rainfall was not significantly correlated with the influenza cases. This association has been suggested in several countries, including Brazil [5], Hong Kong [11], and Senegal [8]. Similarly, some studies (e.g., in Hong Kong [27, 29, 30] and Singapore [27]) have not observed a correlation between influenza cases and rainfall.

Our study suggests an association between suspected influenza cases and some climatic variables in the central and southern regions of Thailand. Therefore, in countries without advanced influenza surveillance systems, appropriate use of ARIMAX models may facilitate the prediction of influenza transmission at present and in the near future. Due to limitations in available and reliable data of influenza time-series in Thailand, the influenza forecasting accuracy is impaired. However, it is hoped that, with further model refinement, more reliable influenza data, and more suitable environmental data (e.g., higher spatial resolution data), these ARIMAX models may provide a sufficiently accurate reference point for public health officials to prepare for and to respond to influenza epidemics.

Admittedly, our study has some limitations. The ARIMA(X) models could only identify correlations, but not causality between influenza cases and average temperature in the central region and between influenza cases and both temperature and average minimum relative humidity in the southern region of Thailand. Consequently, those correlations may act only as proxies for factors not considered in this study. In this analysis, we used suspected influenza cases as a proxy of influenza activity, although it could not stand as a direct measure of influenza morbidity or mortality. However, the suspected influenza cases are sufficient to determine the timing of influenza activity in the central and southern region. The suspected influenza cases are reported only from public hospitals and some private hospitals. The long sampling period may have caused variations in sampling rate due to the laboratory test limitations and changing government policies. Furthermore, this study did not take into account the effects of vaccination on the correlation between influenza cases and the climatic variables [31]. During 2010–2012, approximately 8.18 million influenza vaccines were administered mostly to people of age ≥65 years, people with chronic diseases, and healthcare personnel/poultry cullers [32]. Also, other social and economic parameters were not regarded, which likewise may have played a role in affecting the correlation between influenza activity and climatic variables. Climatic parameters may also affect social behaviors, such as school closure and the tendency for people to stay indoors [33, 34]. Finally, the models did not include vitamin D measurements or solar radiation level data [35, 36].

## Figures and Tables

**Table 1:** The range of climatic parameters in Thailand, 2009–2014.

	Climate factor	Range	Mean ± S.D.
Central	Rainfall	4.0–287.07	149.42 ± 64.6
	Average temperature	24.24–31.6	28.8 ± 1.4
	Maximum relative humidity	66.7–90.6	81.0 ± 4.3
	Minimum relative humidity	37.8–64.0	54.4 ± 6.1
	Average relative humidity	53.4–76.1	65.2 ± 5.0

Southern	Rainfall	18.7–621.0	201.0 ± 86.7
	Average temperature	26.2–29.7	27.8 ± 0.7
	Maximum relative humidity	88.9–96.9	92.5 ± 1.6
	Minimum relative humidity	42.2–70.2	58.5 ± 5.3
	Average relative humidity	68.5–84.3	76.3 ± 3.5

Rainfall in mm, temperature in °C, and relative humidity in percent.

**Table 2:** Central region: cross-correlations between the prewhitened climate and case time-series during 2009–2013.

Variable	Lag				
	0	1	2	3	4
Rainfall	0.346^*∗∗*^	0.391^*∗∗*^	0.344^*∗∗*^	0.363^*∗∗*^	0.286^*∗*^
*P* value	0.0068	0.0022	0.0082	0.0055	0.0325

*T*_mean_	0.007	0.042	0.219	0.440^*∗∗∗*^	0.504^*∗∗∗*^
*P* value	0.9578	0.7508	0.0980	0.0006	<0.0001

RH_max_	0.411^*∗∗∗*^	0.427^*∗∗∗*^	0.247	0.141	0.130
*P* value	0.0011	0.0008	0.0611	0.2940	0.3382

RH_min_	0.498^*∗∗∗*^	0.533^*∗∗∗*^	0.342^*∗∗*^	0.191	0.048
*P* value	<0.0001	<0.0001	0.0086	0.1543	0.7269

RH_mean_	0.460^*∗∗∗*^	0.474^*∗∗∗*^	0.283^*∗*^	0.162	0.081
*P* value	0.0002	0.0001	0.0314	0.2294	0.5551

^*∗*^ *P* < 0.05,  ^*∗∗*^ *P* < 0.01, and ^*∗∗∗*^ *P* < 0.001.

**Table 3:** Southern region: cross-correlations between the prewhitened climate time-series and case time-series during 2009–2013.

Variable	Lag				
	0	1	2	3	4
Rainfall	0.098	−0.118	0.058	0.008	−0.041
*P* value	0.4549	0.3723	0.6643	0.9507	0.7634

*T*_mean_	−0.145	−0.004	0.111	0.281^*∗*^	0.401^*∗∗*^
*P* value	0.2701	0.9741	0.4078	0.0340	0.0022

RH_max_	−0.035	−0.326^*∗*^	−0.017	−0.026	0.140
*P* value	0.7918	0.0118	0.8963	0.8453	0.3049

RH_min_	0.139	0.066	0.147	0.052	−0.120
*P* value	0.2888	0.6183	0.2696	0.6995	0.3775

RH_mean_	0.082	−0.080	0.098	0.032	−0.055
*P* value	0.5335	0.5451	0.4628	0.8106	0.6899

^*∗*^ *P* < 0.05, ^*∗∗*^ *P* < 0.01, and ^*∗∗∗*^ *P* < 0.001.

**Table 4:** Summary of ARIMA model fitting parameters in the central region during 2009–2014.

Model	Fit		Pred.	Climate variables		
	RMSE	AIC	RMSE	Vars	Coef.	*P* value
(1) ARIMA(1, 0, 2)(1, 0, 0)_12_	0.4550	89.74	0.7837			
(2) ARIMAX(1, 0, 2)(1, 0, 0)_12_ with Rainfall	0.4425	88.01	0.7810	Rainfall (lag 0)	−0.1234	0.046
(3) ARIMAX(1, 0, 2)(1, 0, 0)_12_ with Rainfall	0.4420	86.42	0.8339	Rainfall (lag 1)	0.1374	0.0241
(4) ARIMAX(1, 0, 2)(1, 0, 0)_12_ with Rainfall	0.4584	89.99	0.8045	Rainfall (lag 2)	0.0643	0.1989
(5) ARIMAX(1, 0, 2)(1, 0, 0)_12_ with *T*_mean_	0.4344	82.36	0.7042	*T*_mean_ (lag 3)	−0.2806	0.8523
(6) ARIMAX(1, 0, 2)(1, 0, 0)_12_ with *T*_mean_	0.4224	84.13	0.8139	*T*_mean_ (lag 4)	3.9727	0.0356
(7) ARIMAX(1, 0, 2)(1, 0, 0)_12_ with RH_max_	0.4536	91.47	0.7989	RH_max_ (lag 0)	−0.4727	0.6001
(8) ARIMAX(1, 0, 2)(1, 0, 0)_12_ with RH_max_	0.4442	86.95	0.7586	RH_max_ (lag 1)	1.9988	0.0392
(9) ARIMAX(1, 0, 2)(1, 0, 0)_12_ with RH_min_	0.4549	91.73	0.7843	RH_min_ (lag 0)	−0.0469	0.6372
(10) ARIMAX(1, 0, 2)(1, 0, 0)_12_ with RH_min_	0.4170	79.37	0.8880	RH_min_ (lag 1)	2.0353	0.0003
(11) ARIMAX(1, 0, 2)(1, 0, 0)_12_ with RH_min_	0.4439	85.90	0.6760	RH_min_ (lag 2)	−1.3507	0.0256
(12) ARIMAX(1, 0, 2)(1, 0, 0)_12_ with RH_mean_	0.4544	91.59	0.7911	RH_mean_ (lag 0)	−0.2535	0.7034
(13) ARIMAX(1, 0, 2)(1, 0, 0)_12_ with RH_mean_	0.4355	84.52	0.7896	RH_mean_ (lag 1)	1.7995	0.0090
(14) ARIMAX(1, 0, 2)(1, 0, 0)_12_ with RH_mean_	0.4571	89.21	0.7390	RH_mean_ (lag 2)	−0.7104	0.3375
(15) ARIMAX(1, 0, 2)(1, 0, 0)_12_ with *T*_mean_, RH_max_	0.3998	74.98	0.7507	*T*_mean_ (lag 4) RH_max_ (lag 1)	4.4553 2.4223	0.0001 0.0039
(16) ARIMAX(1, 0, 2)(1, 0, 0)_12_ with *T*_mean_, RH_min_	0.3922	70.78	0.9337	*T*_mean_ (lag 4) RH_min_ (lag 1)	3.8566 1.8012	0.0267 0.0026
(17) ARIMAX(1, 0, 2)(1, 0, 0)_12_ with *T*_mean_, RH_min_	0.3786	72.82	0.5792	*T*_mean_ (lag 4) RH_min_ (lag 2)	3.8699 −1.9457	<0.0001 <0.0001
(18) ARIMAX(1, 0, 2)(1, 0, 0)_12_ with *T*_mean_, RH_mean_	0.3786	72.14	0.8027	*T*_mean_ (lag 4) RH_mean_ (lag 1)	4.9256 2.0620	<0.0001 <0.0001

ARIMAX: autoregressive integrated moving average with input series; fit: fitting results; RMSE: root mean square error; AIC: Akaike's Information Criterion; Pred.: prediction of ARIMA model; Coef.: coefficient of climate variables; lag: time lag of climate variables.

**Table 5:** Summary of the ARIMA model fitting parameters in southern region during 2009–2014.

Model	Fit		Pred.	Climate variables		
	RMSE	AIC	RMSE	Vars	Coef.	*P* value
(1) ARIMA(1, 0, 2)(0, 0, 1)_12_	0.3486	57.74	0.2496			
(2) ARIMAX(1, 0, 2)(0, 0, 1)_12_ with *T*_mean_	0.3415	56.65	0.3062	*T*_mean_ (lag 3)	−1.8054	0.6023
(3) ARIMAX(1, 0, 2)(0, 0, 1)_12_ with *T*_mean_	0.3471	56.46	0.2235	*T*_mean_ (lag 4)	2.6311	0.4453
(4) ARIMAX(1, 0, 2)(0, 0, 1)_12_ with RH_max_	0.3480	58.44	0.2748	RH_max_ (lag 1)	−1.5168	0.6160
(5) ARIMAX(1, 0, 2)(0, 0, 1)_12_ with *T*_mean_, RH_max_	0.3483	57.49	0.2589	*T*_mean_ (lag 4) RH_max_ (lag 1)	3.3490 3.6432	0.3580 0.3640

ARIMAX: autoregressive integrated moving average with input series; fit: fitting results; RMSE: root mean square error; AIC: Akaike's Information Criterion; Pred.: prediction of ARIMA model; Coef.: coefficient of climate variables; lag: time lag of climate variables.
